# Validation of the BARD scoring system in Polish patients with nonalcoholic fatty liver disease (NAFLD)

**DOI:** 10.1186/1471-230X-10-67

**Published:** 2010-06-28

**Authors:** Joanna Raszeja-Wyszomirska, Barbara Szymanik, Małgorzata Ławniczak, Maciej Kajor, Alina Chwist, Piotr Milkiewicz, Marek Hartleb

**Affiliations:** 1 Liver Unit, Pomeranian Medical University, Szczecin, Poland; 2 West-Pomeranian University of Technology, Department of Electrical and Computer Engineering, Faculty of Electrical Engineering, Szczecin, Poland; 3 Department of Gastroenterology, Pomeranian Medical University, Szczecin, Poland; 4 Department of Patomorphology, Medical University of Silesia, Katowice, Poland; 5 Department of Gastroenterology and Hepatology, Medical University of Silesia, Katowice, Poland

## Abstract

**Abtract:**

**Background**

Nonalcoholic fatty liver disease (NAFLD) includes a wide spectrum of liver diseases, ranging from pure steatosis to nonalcoholic steatohepatitis (NASH), and eventually to liver cirrhosis with its complications. Identifying advanced fibrosis in patients is crucial to evaluating prognosis and possible therapeutic intervention. A novel, simple, and highly accurate scoring system called BARD, which identifies patients with NAFLD and without significant fibrosis, has been recently introduced and validated in North America..The aim of this study is to validate the BARD scoring system in a Polish cohort with NAFLD.

**Methods**

A group of 104 Caucasians with biopsy-proven NAFLD were included in this study. Fibrosis in liver biopsies was evaluated according to the Histological Scoring System for Nonalcoholic Fatty Liver Disease. The BARD scoring system was assessed according to Harrison et al.: BMI ≥ 28 = 1 point, AST/ALT ratio (AAR) ≥ 0.8 = 2 points, type 2 diabetes mellitus = 1point.

**Results**

Age over 50 and AAR over 0.8 showed, respectively, a moderate and strong association with advanced fibrosis. A BARD score of 2-4 points was associated with F3 or F4 stages of fibrosis with an odds ratio of 17.333 (95% Cl; 3,639 - 82.558) and negative predictive value of 97%.

**Conclusion**

Our results demonstrate that the BARD scoring system has value in the non-invasive diagnosis of advanced fibrosis in NAFLD patients. The vast majority of patients with NAFLD would avoid liver biopsy if BARD was broadly introduced into the clinic.

## Background

Nonalcoholic fatty liver disease (NAFLD) includes a wide spectrum of liver pathologies, ranging from pure steatosis (usually a benign and non-progressive condition) to nonalcoholic steatohepatitis (NASH), which may progress to liver cirrhosis with complications such as portal hypertension and hepatocellular carcinoma [1]. NAFLD is now considered an early expression of the metabolic syndrome [2]. This liver disease is also an independent risk factor for type 2 diabetes mellitus (DMt2) and atherosclerotic cardiovascular events [3].

NAFLD seems to be a growing problem due to the epidemic of obesity. Data from the United States suggests that up to 30% and 3-5% of the general population have NAFLD and NASH, respectively. The prevalence of NASH may attain 25-75% among morbidly obese patients [4]. The natural history of NAFLD shows that histopathological progression occurs in 32-37% of patients over 3-6 years and up to 12% of patients may progress to cirrhosis over 8-10 years [4].

Liver fibrosis, once considered irreversible, is now recognized as a dynamic process with significant likelihood of remission. Therefore, identifying patients with advanced fibrosis is crucial to the accurate evaluation of prognosis, need of surveillance, and therapeutic intervention. The liver biopsy is still the best available standard in the assessment of liver fibrosis. However, liver biopsy has limitations due to invasiveness, small tissue samples, and inter- and intra-observer error. Moreover, liver biopsy is not appropriate to regularly monitor fibrosis progression or response to treatment [4]. Recent evidence indicates that certain demographic and clinical data show relationships with advanced fibrosis in NAFLD patients [5]. On this basis, Angulo et al. [5] proposed a screening algorithm (NAFLD Fibrosis Score) composed of easily obtainable independent indicators of advanced fibrosis, such as age, serum glucose, body mass index (BMI), platelet count, serum albumin, and aspartate aminotransferase/alanine transaminase (AST/ALT) ratio. A scoring system with these 6 variables has an area under the receiver operating characteristic (AUROC) curve of 0.88, with negative predictive value (NPV) of 93% and positive predictive value (PPV) of 90%. NAFLD Fibrosis Score distinguishes patients lacking fibrosis and patients with significant fibrosis, but remains approximately 25% diagnostically uncertain [5]. Other scoring systems, such as the Original European Liver Fibrosis Panel (OELF) or Enhanced Liver Fibrosis (ELF), detect advanced fibrosis. However, their use of parameters not easily available in clinics poses an obvious disadvantage [6]. Negative prediction values of advanced fibrosis for ELF and OELF were 82% and 89%, respectively. Very recently, Harrison et al.[4] proposed the BARD score, which takes into account body-mass index (BMI), AST/ALT ratio (AAR), and presence of type 2 diabetes mellitus (DMt2). These three simple variables were combined in a weighted sum to form a score for predicting advanced fibrosis. A score of 2-4 was associated with an odds ratio for advanced fibrosis of 17 (95% CI: 9.2 to 31.9) and a negative predictive value of 96% [4].

The aim of this study was to validate the BARD scoring system in ethnically homogenous Polish patients with histopathologically diagnosed NAFLD. This study was implanted because the Polish population is formed by a scare number of Afro-Americans and the incidence of obesity is relatively low in confront to the USA figures.

## Methods

The BARD system was retrospectively tested on a cohort of 104 Caucasian patients referred to two Polish liver centers due to elevated liver enzymes and/or hyperintense echo on abdominal ultrasound. Study was approved by the Ethic Committee of Pomeranian Medical University in Szczecin, Poland. Only the patients with biopsy-proven fatty liver (more than 5% of steatotic hepatocytes) who also had a negative history of alcohol intake (i.e., patients consuming less than 20 g/day) were included in this study. All patients tested negative for viral hepatitis B and C (HBs-Antigen and HCV-Antibodies) Patients aged 40 years or less had their ceruloplasmin checked and found normal. Demographic and routine laboratory data were extracted from the database. Liver biopsies were assessed according to the Histological Scoring System for Nonalcoholic Fatty Liver Disease and advanced fibrosis was defined as bridging fibrosis and cirrhosis (F3 and F4).

BARD score was calculated by designating 0-2 points to following parameters: BMI ≥ 28 kg/m^2 ^= 1 point, BMI < 28 kg/m^2 ^= 0 point; AST/ALT ratio ≥ 0.8 = 2 points, AST/ALT ratio < 0,8 = 0 points; freshly recognized or preexisting DMt2 = 1 point. According to original methods, a total of 2-4 points indicates significant fibrosis.

Statistical analyses were performed with χ^2 ^and ANOVA tests using SPSS (Statistical Package for the Social Sciences) version 15.0.1 (evaluation version) statistical software.

## Results

The analyzed cohort consisted of 36 (34.6%) females and 68 (65,4%) males. The median age of patients was 50 years (range in females 30-70 years, in males 23-64 years). BMI in females ranged from 20.0 kg/m^2 ^to 39.3 kg/m^2 ^and in males from 21.9 kg/m^2 ^to 37.7 kg/m^2^. More than half of patients were obese (BMI > 30 kg/m^2^) and 38.1% were overweight (BMI 25-30 kg/m^2^). Tests revealed hypercholesterolemia (>5.18 mmol/L) in 68.9% of patients, hypertriglyceridaemia (>2.03 mmol/L) in 31.2%, DMt2 in 17.8%, and arterial hypertension in 29.7% of individuals. Patients' selected demographic and clinical data are presented in Table 1.

**Table 1:** Clinical characteristics of patients with NAFLD

Variable Normal values	Median	Mean ± SD	Ranges
Age (years)	50	48.0 ± 12.0	26-70

BMI (19-25 kg/m^2^)	29.8	29.6 ± 3.84	21.2-39.3

AST(n = 115) (<38 IU/L)	44.0	51.5 ± 34.9	13-275

ALT(n = 117) (<41 IU/L)	71.0	80.8 ± 55.3	9-290

AST/ALT ratio	0.66	0.79 ± 0.58	0.34-5.23

Glucose (n = 118) (3.89-5.83 mmol/L)	5.44	5.69 ± 1.29	3.44-11.8

Insulin (n = 89) (41.7-187.5 pmol/L)	95.8	152.7 ± 191	13.9-972.3

PLT(n = 118) (150-400 × 10^9^/L)	223	223 ± 62.5	93-376

Albumin (n = 107) (34-48 g/L)	43.0	43.0 ± 4.0	36.0-58.8

HBA_1_c (n = 37) (4.8-5.9%)	5.8	6.2 ± 1.2	4.99-9.6

HOMA (n = 89) (<1.8)	3.4	5.9 ± 7.8	0.44-38.91

Advanced fibrosis was significantly more common in older patients with higher AST/ALT ratios. BMI, gender, and serum AST activity were not significant risk factors for advanced fibrosis in this study. Serum ALT level was lower in patients with F3 and F4 fibrosis stages. Diabetes was twice as frequent in patients with advanced fibrosis, but this difference did not reach clinical significance (*p *= 109). In the population of patients with severe fibrosis (F3-F4) the mean platelet count was significantly lower and mean serum levels of glucose and insulin, as well as HOMA index, were significantly higher in comparison to patients with no or mild fibrosis (F0-F2). No differences were found between serum albumin and glycated hemoglobin levels. The comparative statistics between both groups of patients are summarized in Table 2 and data on relationships between clinical parameters and advanced fibrosis are shown in Table 3. Age ≥ 50 years and AAR ≥ 0.8 were associated with increased risk of severe liver fibrosis. Age ≥ 50 years moderately increased the risk of advanced fibrosis by a factor of 3.455 (95% CI 1.021-11.694). This wide confidence interval depends on the small number of cases and the BARD components seem to be more valuable as less dependent on number of participants. AAR ≥ 0.8 increased advanced fibrosis risk by a factor of 27.

**Table 2:** Comparison of clinical data between patients with no/mild and advanced fibrosis

Variable normal values	Fibrosis 0-2 (n = 88)	Fibrosis 3-4 (n = 15)	*p*
Age (years)	45.0 ± 12.0	53.0 ± 9.0	**P = 0.028**

BMI (19-25 kg/m^2^)	29.5 ± 3.61	30.79 ± 4.90	P = 0.221

AST (<38 IU/L)	52.73 ± 33.36	69.20 ± 45.18	P = 0.097

ALT (<41IU/L)	91.60 ± 56.94	61.53 ± 37.25	P = 0.052

AST/ALT ratio	0.69 ± 0.56	1.32 ± 0.57	**P < 0.001**

Glucose (3.89-5.83 mmol/L)	5.5 ± 1.08	6.99 ± 1.98	P = 0.011

Insulin (41.7-187.5 pmol/L)	132 ± 184	249.3 ± 148.6	P = 0.037

PLT (150-400x10^9^/L)	226.6 ± 61,9	192.3 ± 63.9	P = 0.051

Albumin (34-48 g/L)	43 ± 4.0	43 ± 4.0	P = 0.842

HBA_1_c (4.8-5.9%)	5.8 ± 1.0	7.5 ± 1.8	P = 0.108

HOMA (<1.8)	4.6 ± 6.2	12.2 ± 9.8	P = 0.030

**Table 3:** Risk assessment of clinical parameters for advanced fibrosis

Variable	Fibrosis 0-2 (n = 88)	Fibrosis 3-4 (n = 15)	*p*	OR	95% CI
Age ≥ 50 years	39 (44.3%)	11 (73.3%)	0.038	3.455	1.021-11.694

BMI ≥ 28	58 (65.9%)	10 (66.7%)	0.954	1.034	0.324-3.302

DMt2	14 (15.9%)	5 (33.3%)	0.109	2.643	0.783-8.916

HOMA (<1.8)	32(46.4%)	7(70%)	0.193	2.698	0.644-11.306

AST/ALT ≥ 0,8	17 (19.3%)	13 (86.7%)	p < 0.001	27.147	5.592-131.974

**Figure 1:**
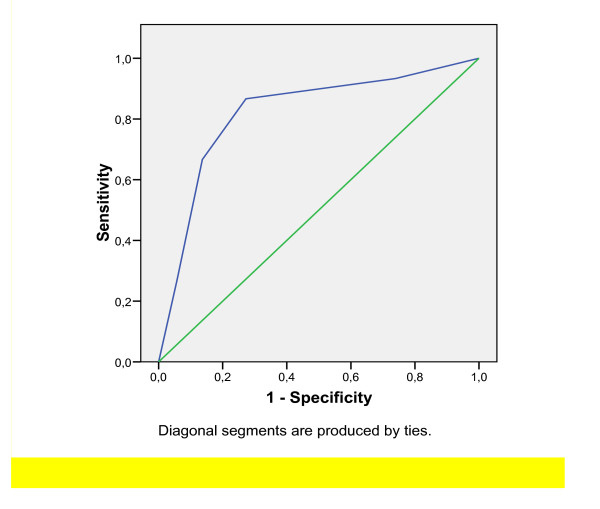
**Simple steatosis plus non-alcoholic steatohepatitis (NASH) with fibrosis stages 0-2 vs. NASH with fibrosis stages 3-4**.

Analysis of the AUROC for BARD was 0.821 (p < 0.001) (see Fig. 1) with an OR of 17.3 (95% CI: 3.64-82.56). The positive and negative predictive values for detecting fibrosis stages F3 or F4 were 35.1% and 97%, respectively. BARD score was characterized by a sensitivity of 86.7% and a specificity 72.7%, when 2 points were set as cut-off value.

## Discussion

The natural history of NAFLD remains poorly understood and the search for noninvasive methods with which to identify patients with advanced fibrosis and cirrhosis remains a key issue. The ideal test for fibrosis in NAFLD has to be simple, amenable to repeated monitoring, inexpensive, reliable, and able to detect the specific degree of fibrosis. Most studies have focused on the identification of severe fibrosis (F3, F4) as these patients have increased risk of hemodynamic or metabolic decompensation and development of liver cancer [7]. Despite the large number of studies (at least 29 since 1999), no simple scoring system that is able to discriminate between severe and no/mild fibrosis in NAFLD patients has been widely popularized.

The present study provides a detailed, descriptive clinical and histopathological analysis of 104 patients with biopsy-proven NAFLD. In this cohort we validated the BARD scoring system, which has recently been developed as a composite score based on the weighted sum of three variables: BMI ≥ 28, AAR ≥ 0.8 and presence of DMt2. These variables were selected as the most useful clinical markers of advanced fibrosis by univariate analysis and forced entry logistic regression performed in a large cohort of 827 patients with NAFLD [4].

The patients analyzed in the present study differ from the patients analyzed in the original study with respect to several important characteristics. First, our analysis is specific to a significantly smaller but ethnically homogenous group of Caucasian patients, diagnosed in two tertiary hepatological centers. Harrison's study [4] comprised an ethnically heterogeneous population in which 32% of patients were non-Caucasian. The ethnic structure of a cohort study is important for any scoring system, as susceptibility to liver steatosis and fibrosis may be related to different genetic and environmental factors. In accordance, Fujii et al.[8] reported significantly worse applicability of BARD in Japanese patients with NAFLD. It is also worthwhile to mention that in Shah et al. study [9] with 541 adults with NAFLD, not racially homogeneous, AUROC for BARD was much lower (only 0.70).

Second, unlike Harrison's study, males dominated in our cohort (66% vs. 49%). Although NASH was initially described as a disease affecting mostly women [10], further studies have not confirmed gender differences in variable NAFLD populations [11].

Third, Polish patients are less obese than the patients participating in the American study. The median BMI in our study was less than 30 kg/m^2 ^compared to 33 kg/m^2 ^in Harrison's work, and no patient presented with morbid obesity (16% of patients in Harrison's group were classified as morbidly obese). Moreover, in our cohort 10% of individuals had normal BMI, compared to 3% in Harrison's group. Regarding body weight, Polish patients had intermediate characteristics between American and Asian populations with NAFLD [4,8]. It is likely that differences in BMI set point, a component of BARD, should be applied to different populations. Although increased BMI is usually found in patients with metabolic syndrome, the central distribution of fat seems to be more important than BMI in the pathogenesis of NAFLD.

Finally, only 18% of Polish patients suffered from DMt2, compared to 35% in Harrison's study [4]. The low number of patients with diagnosed diabetes could also influence our results, as many studies have found that DMt2 is a major predictor of hepatic fibrosis [12].

Marchesini et al.[13] showed that in NAFLD patients a normal or moderately increased body weight and normoglycemia may be associated with clinical and laboratory data similar to those found in obese diabetic patients. The risk of advanced fibrosis in patients with abnormal HOMA (>1.8) was increased by a factor of 2.7 in our study. This finding strongly suggests that insulin resistance is a key factor triggering fibrosis. Underestimation of DMt2 in our study is an argument for advising oral glucose tolerance tests as a routine strategy in the assessment of patients with NAFLD [14].

Despite major differences in the two populations, we obtained very similar results to Harrison's work, indicating that BARD has high predictive value in the diagnosis of advanced fibrosis. In Polish NAFLD patients a BARD score of 2-4 was associated with an OR of 17.33 of advanced fibrosis. The composite score for sensitivity and specifity, as determined by AUROC in our study, was equivalent to the originally described score (0.82 vs. 0.81, respectively). Similarly, in our study negative prediction of advanced fibrosis (NPV 97%) was much better than its positive prediction (PPV 35.1%). In other words, BARD's results yielded a very low number of false negative and high number of false positive diagnoses of significant fibrosis. Negative prediction of fibrosis in NAFLD patients was better than using ELF or OELF scoring systems (82% and 88%, respectively), based on non-routine laboratory data [6]. In our study, BARD would permit avoidance of liver biopsies in 100 of 104 patients who did not require this procedure.

Our results, similar to originally described, were obtained in smaller population of patients with NAFLD. However the results of Shah et al.[9] were different despite the number of participants: 541 vs 827 in Harrison et al. work [4]. It is of importance to note down that the AUROC for the NAFLD Fibrosis Score was also lower in Shah's [9] study than in original Angulo et al. [5] paper and the FIB4 with the AUROC of 0.8 was superior of the other noninvasive panels tested this work. Studies mentioned above demonstrated utility of noninvasive panels to stage liver disease as well as their limitation. The first limitation of all these studies is considerable sampling variability in fibrosis staging in liver biopsies and the second is the analyzed cohort with a broad distribution of fibrosis stage and low number of patients with advanced fibrosis. It is also important to notice that neo-fibrogenesis is present in fatty liver, as showed by Tarantino et. al [15]. The results of our study showed that the BARD scoring system is useful despite moderate number of participants with severe fibrosis. It seems that improving BARD's diagnostic sensitivity would necessitate incorporation of the markers of subclinical portal hypertension (e.g., platelet count) and hepatic synthetic capacity (e.g., albumin). This conclusion is justified by the satisfactory positive prediction of advanced fibrosis by the NAFLD fibrosis scoring system proposed by Angulo et al. [5]. This is also the idea of searching the perfect test to replace liver biopsy.

In populations of patients with more advanced fibrosis, univariate analysis revealed that age ≥50 and AAR ≥ 0.8 were risk factors of severe liver fibrosis. The mean AAR in patients with advanced fibrosis was 1.32, definitely the strongest predictor of advanced fibrosis (OR: 27.15). Interestingly, high AAR resulted from both the lower levels of ALT and higher levels of AST in the subgroup of patients with significant fibrosis. There are several hypotheses explaining an influence of progressive fibrosis on aminotransferase activity, but none have been fully validated.

An ideal noninvasive test for assessment hepatic fibrosis would be one that is sensitive, specific, free of additional cost to the patient, and applicable across all chronic liver diseases. The BARD scoring system is one of the attempt to create such a score although an ideal test does not exist till now.

## Conclusion

BARD reliably identifies patients without significant fibrosis, providing the opportunity to avoid liver biopsy in a large proportion of patients with NAFLD. This simple scoring system shows utility and applicability to Caucasian populations with NAFLD.

## Competing interests

The authors declare that they have no competing interests.

## Authors' contributions

JRW wrote the manuscript, BS performed the statistical analysis, MŁ and AC were responsible for database, MK evaluated liver biopsies, PM participated in the desing of the study and revising the manuscript, MH was responsible for coordination and revising the manuscript. All authors read and approved the final manuscript.

## Pre-publication history

The pre-publication history for this paper can be accessed here:

http://www.biomedcentral.com/1471-230X/10/67/prepub
